# Special Issue: Corrosion Technology and Electrochemistry of Metals and Alloys

**DOI:** 10.3390/ma19163448

**Published:** 2026-08-14

**Authors:** Seunghyun Kim

**Affiliations:** Department of Nuclear Safety Research, Korea Institute of Materials Science (KIMS), 797 Changwon-daero, Seongsan-gu, Changwon-si 51508, Republic of Korea; skims@kims.re.kr

Few degradation phenomena are as universal—or as expensive—as the corrosion of metals and alloys: recent global estimates put its annual cost at some USD 2.5 trillion, more than 3% of world GDP [1]. Because corrosion is at heart an electrochemical process, occurring wherever a metallic surface exchanges charge with its environment, progress in understanding and controlling it has always been tied to progress in electrochemistry itself. That connection has deepened considerably in recent years. Electrochemical impedance spectroscopy (EIS), potentiodynamic polarization, and linear polarization resistance have matured into a quantitative toolbox [2]; surface-analytical techniques now resolve passive films down to near-atomic scales [3]; and mechanistic models increasingly convert electrochemical data into predictions of oxide growth and metal release, rather than mere rankings of materials.

Substantial knowledge gaps nevertheless remain. Laboratory measurements span hours to weeks, whereas the components they are meant to qualify must survive years or decades of service. Mechanistic models are usually validated under fixed conditions, whereas real environments—such as power-plant coolants whose chemistry evolves over an operating campaign, molten salts subjected to daily thermal cycling, and fermentation media teeming with microorganisms—are coupled and time-dependent. Moreover, mitigation strategies that look promising at coupon scale must still demonstrate their durability, scalability, and cost at component scale.

This Special Issue, “*Corrosion Technology and Electrochemistry of Metals and Alloys*,” was conceived as a forum for work that narrows these gaps by combining careful electrochemical measurement with surface and microstructural analysis, and, wherever possible, quantitative interpretation. Following rigorous peer review, seven manuscripts were published, contributed to by research groups from eight countries across Europe and Asia, and the collection has since been viewed nearly nineteen thousand times—a level of readership that reflects the breadth of interest in the topic. The published articles, listed at the end of this Editorial, fall into four connected themes.

The first theme concerns alloy corrosion in demanding high-temperature service. Bojinov, Betova, and Karastoyanov studied Alloy 690 steam-generator tubing in simulated primary coolant, combining in situ impedance spectroscopy at 300 °C and 9 MPa with X-ray photoelectron spectroscopy and a new version of the mixed-conduction model to quantify how oxide growth and corrosion release respond to the evolution of the coolant chemistry over an operating campaign [4]—a question of direct industrial relevance as water-cooled plants weigh alternatives to enriched lithium hydroxide for pH control. Shin and co-workers examined super duplex stainless steel AISI 2507 at 700 °C, the ignition temperature adopted for lithium-ion battery cases. Internal cell temperatures approaching 870 °C have indeed been measured during thermal runaway of large-format cells [5]. They showed that secondary phases precipitate within a few hours at this temperature and set up galvanic couplings that depress the open-circuit potential and raise the corrosion current density, making secondary-phase control a prerequisite for the high-temperature safety of battery enclosures [6].

The second theme is mitigation through surface engineering. The molten carbonates proposed for thermal energy storage in next-generation concentrated solar power plants rank among the most aggressive environments the energy transition has produced [7]. Morales, Rezayat, and Mateo introduced, for the first time, an amorphous carbon film as a protective surface modification for stainless steel in these salts. During 1000 h at 600 °C, the 100 nm film decomposed into iron- and manganese-rich carbides that promoted denser, better-adhered oxide scales, hindered Li^+^ diffusion, and lowered the corrosion rate by about 20%—evidence that even a nanometric surface treatment can measurably extend component life in an extreme environment.

The third theme reverses the perspective: the anodic dissolution that corrosion science works to suppress can, once brought under control, serve as a manufacturing tool [8]. Kowalik and colleagues evaluated novel organic additives for copper electrorefining and showed that pairing a new polyhexamethylene-biguanidine-based ionic liquid with thiourea achieves a current efficiency of 97% together with low cell voltage, low specific energy consumption, and smoother cathode deposits than the industrial bone-glue/thiourea standard. Qin and co-workers, in turn, exploited the strong passivating action of nitrate ions to localize the anodic dissolution of the nickel-based superalloy GH4169 (Inconel 718) in roll-print mask electrochemical machining, producing well-defined micro-pit arrays and identifying the process window that optimizes their profile and localization.

The fourth theme carries electrochemistry into real service environments, where idealized laboratory media give way to chemically and biologically complex conditions. Dimitrijević and colleagues compared copper and Cu72Zn28 brass in apricot fermentation liquid, an aggressive and biologically active medium encountered by distillation equipment. Open-circuit potential, linear polarization resistance, and Tafel measurements showed that copper corrodes at a current density 3.8 times lower than that of brass, confirming it as the more suitable material, while the study also highlights the role of microbiologically influenced corrosion—a phenomenon whose mechanisms remain only partially resolved [9]—in the degradation of such equipment in the field.

Different as these systems are, the seven studies share a methodological backbone—electrochemical measurement combined with surface and microstructural characterization and, in several cases, quantitative modelling—and it is from this shared foundation that the most promising directions for future research emerge.

First, long-duration and in situ monitoring should become routine. Several of the studies in this issue make the gap explicit: meaningful qualification requires behavior projected over operating campaigns and decades of service, yet most electrochemical data are still collected over hours to weeks. Standardized high-temperature, high-pressure in situ impedance measurements, together with longer controlled exposures, are needed to close the distance between laboratory and service timescales. Second, mechanistic frameworks such as the mixed-conduction and point-defect models [10] should be generalized from fixed test conditions to the coupled, time-evolving chemistries of real systems. Moreover, they should be increasingly combined with data-driven and machine-learning methods capable of exploiting large electrochemical datasets for predictive corrosion assessment [11]. Third, protective surface treatments, including amorphous carbon films, warrant systematic investigation of their long-term durability, self-healing capability, scalability, and cost, so that proof-of-concept results can mature into deployable technologies.

Fourth, the community should continue extending its attention to the emerging extreme environments associated with the energy transition—including evolving power-plant water chemistries, carbonate and chloride thermal-storage salts, and the high-temperature interior of battery systems—where established passivity rules may no longer hold. Fifth, microbiologically influenced corrosion and other real-service phenomena demand experimental designs that faithfully reproduce multifactorial, biologically active media, so that material rankings obtained in clean electrolytes survive contact with the field. Finally, as electrochemical processing becomes central to a circular, low-carbon economy, the efficiency and durability of operations such as electrorefining and electrochemical machining should be optimized hand in hand with sustainability goals. Progress along all of these lines will require sustained collaboration among electrochemistry, materials science, surface analysis, and computational modelling.

As Guest Editor, I hope that this Special Issue offers readers a coherent cross-section of contemporary corrosion technology and electrochemistry, and that it stimulates further work along the directions outlined above. I am deeply grateful to all of the authors for their high-quality contributions and to the reviewers whose careful, constructive comments improved the published works. The success of this Special Issue would not have been possible without the dedication of the editorial staff of *Materials*, and in particular of the Section Managing Editor, Ms. Serena Shi, whose support throughout the process is gratefully acknowledged.
